# Effects of Systemic Lupus Erythematosus on Clinical Outcomes and In-Patient Mortality Among Hospitalized Patients With Diverticulitis

**DOI:** 10.7759/cureus.26603

**Published:** 2022-07-06

**Authors:** Ahmed Ahmed, Amjad Shaikh, Yasir Rajwana, Sushil Ahlawat

**Affiliations:** 1 Gastroenterology and Hepatology, Rutgers University New Jersey Medical School, Newark, USA; 2 Internal Medicine, Rutgers University New Jersey Medical School, Newark, USA; 3 Internal Medicine, Jersey City Medical Center, Jersey City, USA

**Keywords:** systemic lupus erythematosus, chronic inflammation, colorectal disease, autoimmune disease, lupus, diverticulitis

## Abstract

Purpose

Though there are studies on other autoimmune diseases, the literature is deficient on the associations between systemic lupus erythematosus (SLE) and diverticulitis. This study aims to evaluate the effects of SLE on clinical outcomes and in-patient mortality in patients with diverticulitis.

Methods

The National Inpatient Sample (NIS) database was used to identify adult patients with diverticulitis-related hospitalizations from 2012 to 2014 using the International Classification of Diseases, Ninth Revision, Clinical Modification (ICD-9-CM) codes. Primary outcomes were mortality, hospital charges, and length of stay (LOS). Secondary outcomes were effects on the complications associated with diverticulitis. Chi-squared tests and independent t-tests were used. Multivariate analysis was performed to assess the primary outcomes after adjusting for confounding variables.

Results

There were 2,553,320 diverticulitis-related hospitalizations from 2012 to 2014, of which 13,600 patients had SLE. The average LOS was 5.2 days, mortality rate was 0.8%, and total hospital charges per patient were $43,970. SLE was associated with a statistically significant longer LOS and higher hospital costs. SLE was statistically significant for having higher perforation rates but lower rates for morality, abscesses, and fistula formation. Differences in complications such as sepsis, gastrointestinal bleeding, and surgical intervention requirement were non-significant.

Conclusion

Since SLE causes a high inflammatory state, one would expect higher rates of complications and possibly higher mortality rates in those with concomitant diverticulitis. However, although there was a higher LOS and hospital cost, the mortality rate was lower and only a complication of perforation was found to be higher in SLE patients.

## Introduction

Systemic lupus erythematosus (SLE) is an autoimmune disease that can affect any organ system and is characterized by a high inflammatory state. Studies have estimated that the global incidence of SLE ranges from 0.3 to 23.2 per 100,000 per year, though, within the US, the estimated frequency of SLE appears to be increasing with increased prevalence in females and African American, Asian, and Hispanic American populations [1-3]. SLE induces a multisystemic inflammatory response that commonly leads to complications such as accelerated coronary artery disease, end-stage renal disease, dermatological scarring, and neuropsychiatric comorbidities [3]. The gastrointestinal (GI) tract is also frequently affected and can manifest as processes such as pancreatitis, mesenteric vasculitis, and liver diseases such as autoimmune hepatitis and primary biliary cirrhosis [4-8].

Although the mortality rate of SLE has been improving over the past few decades, mortality relative to other autoimmune diseases remains high [2]. Moreover, SLE continues to be one of the top 20 leading causes of death in females aged five to 64 years [1,2]. Given the complex inflammatory state of SLE, patients may experience multiorgan involvement leading to high morbidity, with one study demonstrating that the mortality rate attributed to SLE may be underreported due to the various other complications that may result from the disease [2]. SLE patient mortality is frequently associated with cardiovascular disease, cancer, infections, and renal disease [4-8].

Diverticular disease is one of the most common manifestations of GI disorders [9,10]. Diverticulitis occurs when a diverticulum becomes infected or inflamed causing symptoms such as pain and hematochezia. The incidence of diverticulitis has increased over the last several decades, with an estimation of more than 2.7 million out-patient visits and 200,000 in-patient admissions per year in the US [9-13]. In particular, the prevalence of diverticulitis in younger populations has substantially increased [12,13]. Though relatively rare, diverticulitis can result in several complications. In one population-based study, approximately 12% of patients with diverticulitis developed complications associated with the disease, with the most common complications being abscess formation, peritonitis, obstruction, and fistula formation [9,13]. Additionally, diverticulitis may reoccur, with one UK-based study finding that 11.2% of patients admitted for acute diverticulitis were admitted again for recurrence [14].

While the complications and mortality of these two diseases have been studied independently, the literature is deficient on the associations between SLE and diverticulitis. This study aimed to evaluate the effects of SLE on clinical outcomes and in-patient mortality in patients with diverticulitis.

## Materials and methods

Data source

The Healthcare Cost and Utilization Project (HCUP) National Inpatient Sample (NIS) is the largest national all-payer inpatient database, which contains data on more than seven million hospital stays [14,15]. The NIS was queried from 2012 to 2014 using the International Classification of Diseases, Ninth Revision, Clinical Modification (ICD-9-CM) codes to identify hospitalized patients with diverticulitis who had SLE (Table 1).

**Table 1:** Appendix of the International Classification of Diseases, Ninth Revision (ICD-9) codes

Diagnosis	ICD-9 code
Systemic lupus erythematosus	710.0
Diverticulitis	562.10, 562.11, 562.12, 562.13, 562.03, 562.02, 562.01
Obstruction	560.9, 997.49, 560.8, 560.81, 537.3
Bowel perforation	569.83
Abscess	682.9, 567.22, 566, 569.5
Bowel surgery	45.60-45.63, 45.7-45.79, 45.8-45.83
Abscess	682.9, 567.22, 566, 569.5
Sepsis	995.92, 995.91
Fistula	686.9, 569.81
GI bleed	578.9, 459.0
Fistula	686.9, 569.81

The NIS is a product of the Agency for Healthcare Research and Quality and contains de-identified patient information. The data used are a nationally representative subset obtained through hospital discharge records. Though a proportion of the national population has been sampled, a yearly sampling weight has been applied, which provides adequate national estimates. Many studies have verified the value of this sampling tool, and, therefore, it was used for this study. Given that NIS data are de-identified, Institutional Review Board (IRB) approval was not obtained.

Study design and inclusion criteria

This was a cross-sectional study and included all patients aged > 18 years with a primary diagnostic code for diverticulitis from 2012 to 2014. The ICD-9-CM codes used were 562.10, 562.11, 562.12, 562.13, 562.03, 562.02, and 562.01. The database was then queried to include all patients with a diagnosis of SLE (710.0). Patients included in the study were required to have a primary diagnosis of diverticulitis. Included patients were then divided into two groups, those with and without SLE. Primary outcomes measured were in-patient mortality, hospital charges, and length of stay (LOS). Secondary outcomes were diverticulitis-related complications, including perforation, obstruction, GI bleeding, fistula/abscess formation, sepsis, and the requirement for surgical intervention. Various patient demographics (age, race, sex, income, and insurance status) and comorbidities were obtained. The severity of the comorbidities was analyzed via the Deyo modification of the Charlson Comorbidity Index (CCI). CCI measures 17 common medical conditions and assigns different weights to develop a score from 0 to 33, which is then used to correlate the overall severity of illness.

Statistical analysis

All statistical analyses were performed using IBM SPSS Statistics version 26 (IBM Corp., Armonk, NY). Chi-squared tests and independent t-tests were used to compare outcomes for categorical and continuous data, respectively, between the two groups. A multivariate logistic regression model was designed to investigate the associations between diverticulitis complications and SLE. The hierarchical model included both patient characteristics (age, race, sex, and comorbidities) and the CCI. To limit the effect of cofounders, this was the primary means by which adjustments were made in the data for patient characteristics. Univariate analysis was conducted on the aforementioned factors. Complicated diabetes mellitus, anemia, renal failure, cardiovascular disease, congestive heart failure, age, race, insurance status, and sex were included in the multivariate analysis with P < 0.05 indicating statistical significance. Adjusted odds ratios were calculated for each primary outcome with 95% confidence intervals (CIs).

## Results

There were 2,553,320 diverticulitis-related hospitalizations from 2012 to 2014, of which 13,600 patients had SLE. In patients with SLE and diverticulitis, 90% (12,265) were females compared to 57.7% (1,464,175) in those without SLE. SLE patients were also found to be younger with an average age of 62.5 vs. 68.5 years in the control group. Moreover, non-SLE patients were more likely to be Caucasian at 76.2% vs. 62.9% (Table 2).

**Table 2:** Demographics and resource utilization of diverticulitis patients with and without lupus

	Diverticulitis without lupus (N = 2,539,720)	Diverticulitis with lupus (N = 13,600)	P-value	95% CI
Mean age (years)	68.5 (15.0 SD)	62.5 (13.6 SD)	<0.05	5.78-6.28
Sex			<0.05	
Female	1,464,175 (57.7%)	12,265 (90.2%)		
Male	1,075,025 (42.3%)	1,335 (9.8%)		
Race			<0.05	
White	1,849,365 (76.2%)	8,210 (62.9%)		
Black	271,285 (11.2%)	2,940 (22.5%)		
Hispanic	210,785 (8.7%)	1,405 (10.8%)		
Asian or Pacific Islander	32,695 (1.3%)	115 (0.9%)		
Native American	10,660 (0.4%)	65 (0.5%)		
Others	52,740 (2.2%)	325 (2.5%)		
Length of stay in days	4.9 (5.1 SD)	5.2 (4.4 SD)	<0.05	-0.37 to -0.20
Total charges	$41,713 (57,201 SD)	$43,970 (63,293 SD)	<0.05	-3235 to -1280
Charlson Comorbidity Index	3.7 (2.3 SD)	4.1 (2.0 SD)	<0.05	-0.41 to -0.34

In SLE patients, the average LOS was found to be longer (5.2 days vs. 4.9 days) and the mortality rate was lower (0.8% vs. 1.2%), and they had higher total hospital charges ($43,970 vs. $41,713). SLE was associated with a statistically significant longer LOS, higher hospital costs, higher CCI (4.1 vs. 3.7), and a lower mortality rate. In terms of clinical outcomes, SLE was statistically significant for having higher perforation rates (36.9% vs. 35.8%, OR: 1.05, 95% CI: 1.01-1.09) and lower rates for abscess (4.4% vs. 5.1%, OR: 0.87, 95% CI: 0.80-0.94) and fistula formation (0.3% vs. 0.5%, OR: 0.68, 95% CI: 0.51-0.91) (Table 3).

**Table 3:** Clinical outcomes in diverticulitis patients with and without lupus

	Diverticulitis without lupus (N = 2,539,720)	Diverticulitis with lupus (N = 13,600)	P-value	Odds ratio
GI bleed	118,535 (4.7%)	600 (4.4%)	0.16	0.94
Perforation	909,855 (35.8%)	5,020 (36.9%)	<0.05	1.05
Abscess	128,545 (5.1%)	600 (4.4%)	<0.05	0.87
Obstruction	101,440 (4.0%)	445 (3.3%)	<0.05	0.81
Surgical intervention (colectomy)	168,150 (6.6%)	860 (6.3%)	0.16	0.95
Fistula	12,305 (0.5%)	45 (0.3%)	<0.05	0.68
Sepsis	121,855 (4.8%)	635 (4.7%)	0.48	0.97
In-patient mortality	30,005 (1.2%)	105 (0.8%)	<0.05	0.65

Differences in complications such as sepsis, GI bleeding, and requirement for surgical intervention were not found to be significant.

## Discussion

In this study, we hypothesized that the pan-inflammatory state of SLE would exacerbate complications in patients with concurrent diverticulitis and thus lead to adverse outcomes such as prolonged hospitalization, increased hospital charges, and higher mortality. Compatible with the hypothesis, the data in this study showed that SLE was associated with statistically significant higher perforation rates, a longer LOS, and higher hospital costs. However, contrary to the hypothesis, the data demonstrated a lower mortality rate and decreased rates of abscess and fistula formations in SLE patients with concomitant diverticulitis. Additionally, while patients with SLE had statistically longer LOS and hospital costs, the difference may not be clinically significant due to the relatively small difference. Since SLE is known to cause systemic damage and have a profound inflammatory state, it is important to understand the associations found in this study, as it can help identify high-risk patients and possibly prevent morbidity and mortality in such cases.

Diverticulitis is associated with rare complications that can occur as part of its natural disease course. Such complications include abscess formation and perforation. The underlying cause of diverticulitis itself is micro- or macroscopic perforation of a diverticulum, which are points of weakness corresponding to where the vasa recta penetrate the colon's circular muscle layer. The literature is versed in the pathogenesis of diverticula, with abnormal colonic motility being an important predisposing factor [16]. It is hypothesized that the increase in intraluminal pressure predisposes to herniation of mucosa and submucosa. As a diverticulum herniates, the penetrating vessel responsible for the wall weakness becomes more exposed and susceptible to injury and eventual rupture. Erosion of the diverticular wall by the increased intraluminal pressure and digested food lead to focal necrosis, inflammation, and possible perforation [16]. Although only 1-2% of patients with acute diverticulitis have a perforation with purulent or fecal peritonitis, mortality rates can be as high as 20% [17,18].

Though the association between SLE and diverticulitis has not been previously explored in the literature, it is well established that both disease processes can have their own associated complications within the GI tract. As alluded to before, SLE involves a pan-inflammatory state affected by multifactorial pathways, including genetic, environmental, immunoregulatory, hormonal, and epigenetic variables [18]. Such variables lead to interactions that promote cytokine release, autoantibody formation, immune complex deposition, and autoreactive T-cell activation [19]. SLE is traditionally associated with renal, cardiac, and hematological abnormalities, but the involvement of the GI tract is not uncommon [20]. Within the GI tract, SLE has a diverse presentation, including bowel ulcerations, mesenteric vasculitis, pancreatitis, SLE-associated hepatitis, and primary biliary cirrhosis [21].

In general, autoimmune diseases, including rheumatoid arthritis, SLE, and Behçet's disease, typically involve inflammatory processes that can cause changes to the endothelium of small arteries and capillaries, leading to ischemia and necrosis in any part of the GI tract, resulting in ulceration, perforation, or even hemorrhage [22]. Even though the exact etiology of SLE remains ambiguous, it is well documented in the literature that many of the clinical manifestations of SLE are mediated directly or indirectly by antibody formation and the creation of immune complexes (IC). In terms of GI manifestations, the pathogenesis of SLE-related intestinal pseudo-obstruction is still preliminary and being studied, but possible mechanisms have been theorized to include immune complex deposition in smooth muscle cells, and vasculitis leading to chronic ischemia and hypomotility [23]. Moreover, SLE patients may develop mesenteric thrombosis or pancreatitis with the proposed pathogenesis of vasculitis as a consequence of immune complex deposition and thrombosis of the small intestinal vessels due to circulating antiphospholipid antibodies [24]. Though rare, mesenteric thrombosis can ultimately cause bowel wall perforation or even infarction. In accordance with the literature, studies have found that patients with SLE are independently at risk of intestinal perforation [25,26]. Such a process of hypomotility, profound immune complex deposition, and vasculitis processes can lead to localized inflammation and wall weakness, which are known risk factors for the development of diverticula and the possible complication of perforation. The data in this study are compatible with the literature, as the perforation rates were found to be higher in SLE patients, which is likely a consequence of the inherent risk of GI perforations associated with the disease.

While SLE itself may not necessarily directly impart an increased risk of diverticulitis, the treatment regimen may predispose the patient to diverticulitis and its various complications. Nonsteroidal anti-inflammatory drugs (NSAIDs), a common treatment regimen in SLE, have been shown to be associated with diverticulitis and increased risk of diverticular perforation [27-30]. Moreover, studies have found that chronic NSAID use is not only associated with but leads to higher rates of complications in diverticulitis [31]. Corticosteroid use, another common therapeutic in SLE, has also been shown to increase the risk of diverticular perforation [32,33]. Moreover, one study of patients, including those with autoimmune diseases, on immunosuppressive therapies demonstrated a higher risk of colonic perforation and longer hospital stays [34]. Though the literature has demonstrated that biologics such as tocilizumab can have an association with diverticulitis and perforation, one study following rheumatoid arthritis patients demonstrated that the increased risk of GI perforation was only statistically significant when a biologic was concurrently used with glucocorticoids [35]. SLE confers an inherent risk of perforation, yet we posit that common SLE treatments such as NSAIDs, biologic therapies, and steroid use also increase this risk. It is, therefore, likely that the results of this study, which demonstrated that SLE patients had higher rates of perforation, are due to complex multifactorial causes. Moreover, this study is compatible with the literature as the LOS and hospital costs were found to be higher compared to the control group. SLE patients are immunocompromised and thus at higher risk of complications, which would theoretically lead to longer LOS, more procedures and therapies, and, thus, higher hospital costs. Moreover, the higher CCI in SLE patients was statistically significant, further demonstrating the complexity in these patients, which would likely increase their hospital stay and risk of developing complications such as perforations.

The difference in the rates of development of fistulas can likely be explained by the underlying disease process. Research in patients with inflammatory bowel disease has demonstrated that the formation of fistulas requires persistent inflammatory triggers to initiate and sustain the necessary molecular and pathophysiological processes [36]. Given that SLE is associated with a sustained state of inflammation, one would assume higher rates of fistulas in such patients. On the contrary, the data in this study were notable as they showed lower rates compared to the control. We posit that immunotherapies involved in the treatment of SLE play a role in such findings. Glucocorticoids and disease-modifying antirheumatic drugs (DMARDs) are known to suppress the immune system, and we theorize that the local environment of cytokines and growth factors needed for the inflammatory changes leading to fistulas may be diminished in well-controlled SLE patients.

In terms of the rates of abscess formation, we hypothesized higher rates in SLE patients, yet the opposite effect was found. Given the fact that SLE patients are immunocompromised and likely to have other underlying comorbidities, as demonstrated by a higher CCI, we postulate that SLE patients are more likely to receive broader spectrum antibiotics earlier in their hospital course. This bias may lead SLE patients to receive more aggressive therapies, imaging, and procedures earlier than non-SLE patients and ultimately affect the development of more severe infections such as abscesses.

Since SLE causes a high inflammatory state, one would expect higher rates of complications and possibly higher mortality rates in those with concomitant diverticulitis. However, the mortality rate in this study was found to be lower. This was a notable finding since the risk of perforation was found to be higher in SLE patients with diverticulitis. A nationwide retrospective Canadian study of diverticulitis prognosis demonstrated an in-hospital mortality rate of 0.4% in the ages between 51 and 60 years and 3.3% in patients above 60 years [37]. The data in our study demonstrated a lower mortality rate, with the mean age being approximately 68 years in non-SLE patients and 62 years in SLE patients.

Similar to other studies, perforation from diverticulitis with fecal peritonitis was the highest risk of mortality, with some studies reporting the risk to be around 45-50% when compared to purulent perforations [36-38]. We speculate that the majority of the perforations recorded for SLE patients were purulent, which tends to have a lower mortality rate compared to feculent perforations [38,39]. Moreover, as alluded to before, we postulate that SLE patients are likely to receive more aggressive therapies and diagnostic imaging, which would affect the timing of diagnosis and treatment of complications, such as a perforation, which would ultimately affect mortality outcome results.

Given that the NIS database is developed using billing code data, there are some limitations to this study. Foremost, lab data that would assist in assessing the severity of the disease are not included, so it is difficult to determine on a case-by-case basis. Elixhauser comorbidity scores are used to assist in determining the baseline level of health of the patient using weighted scores from multiple medical conditions, which may help to ameliorate this. However, this would not necessarily indicate the severity of a patient’s underlying SLE. Additionally, given that individual medical treatments are not input via ICD-9 codes in this database, it is also difficult to delineate the exact treatment each patient received or what medications were given. Given these limitations, further studies, including patient lab data and treatment plans, would allow further assessment of the effects of SLE on pancreatitis.

## Conclusions

Since SLE is known to develop a high inflammatory state, one would expect higher rates of complications and possibly higher mortality rates in those with concomitant diverticulitis. In this study, though the primary outcomes were significant, the mortality rate was actually lower, and only perforation was found to be higher in SLE patients. A high inflammatory state likely increases the risk of perforation and LOS in SLE patients, yet factors such as glucocorticoid/anti-inflammatory use can be a contributing factor to such complications, as well as to the mortality rate. This study identifies a lower mortality rate but a higher LOS and hospital cost association between diverticulitis and SLE, and future studies are needed to further analyze this association.
